# The effects of educational curricula and training on LGBT-specific health issues for healthcare students and professionals: a mixed-method systematic review

**DOI:** 10.7448/IAS.20.1.21624

**Published:** 2017-07-19

**Authors:** Adekemi Oluwayemisi Sekoni, Nicola K. Gale, Bibiane Manga-Atangana, Arjun Bhadhuri, Kate Jolly

**Affiliations:** ^a^ Department of Community Health and Primary Care, College of Medicine, University of Lagos, Lagos, Nigeria; ^b^ Health services management Centre, University of Birmingham, Birmingham, UK; ^c^ Institute of Applied Health Research, University of Birmingham, Birmingham, UK

**Keywords:** systematic review, LGBT health, education, healthcare students, healthcare professionals

## Abstract

**Introduction**: Poor access of lesbian, gay, bisexual and transgender (LGBT) people to healthcare providers with clinical and cultural competency contributes to health inequalities between heterosexual/cisgender and LGBT people. This systematic review assesses the effect of educational curricula and training for healthcare students and professionals on LGBT healthcare issues.

**Methods**: Systematic review; the search terms, strategy and process as well as eligibility criteria were predefined and registered prospectively on PROSPERO. A systematic search of electronic databases was undertaken. Screening for eligible studies and data extraction were done in duplicate. All the eligible studies were assessed for risk of bias. The outcome of interest was a change in participants’ knowledge, attitude and or practice.

**Results**: Out of 1171 papers identified, 16 publications reporting 15 studies were included in the review. Three were non-randomized controlled studies and 12 had a pre/post-design; two had qualitative components. Bias was reported in the selection of participants and confounding. Risk reported was moderate/mild. Most studies were from the USA, the topics revolved around key terms and terminology, stigma and discrimination, sexuality and sexual dysfunction, sexual history taking, LGBT-specific health and health disparities. Time allotted for training ranged from 1 to 42 hours, the involvement of LGBT people was minimal. The only intervention in sub-Saharan Africa focused exclusively on men who have sex with men. All the studies reported statistically significant improvement in knowledge, attitude and/or practice post-training. Two main themes were identified from the qualitative studies: the process of changing values and attitudes to be more LGBT inclusive, and the constraints to the application of new values in practice.

**Conclusions**: Training of healthcare providers will provide information and improve skills of healthcare providers which may lead to improved quality of healthcare for LGBT people. This review reports short-term improvement in knowledge, attitudes and practice of healthcare students and professionals with regards to sexual and LGBT-specific healthcare. However, a unified conceptual model for training in-terms of duration, content and training methodology was lacking.

## Introduction

Globally, there is dearth of information on the health of lesbian, gay, bisexual and transgender (LGBT) people. However, available evidence from different regions of the world has consistently demonstrated poorer health outcomes among LGBT people compared to the general population [1–3]. Discrimination in healthcare settings against LGBT people can manifest as outright denial of care, disrespect and abuse, low-quality care, negative attitude and behaviour of providers, and lack of confidentiality and privacy in service provision [4].

In the case of HIV/AIDS, discrimination has resulted in barriers to accessing HIV prevention services, HIV and sexually transmitted infection (STI) testing services, enrolment and retention in treatment and care and support programmes for people infected with HIV [5,6]. Over the past decade, significant improvement has been made in the global effort at slowing the pace of the HIV epidemic. The incidence and prevalence of HIV infection and HIV-related mortality has dropped [7]. A high proportion of people living with the virus are on life-saving antiretroviral drugs and achieving good health [8,9]. However, in many regions of the world, the burden of new HIV infection is disproportionately higher among men who have sex with men (MSM), transgender women, people who inject drugs and sex workers [10]. Despite the overall progress, these disparities suggest that the goal of achieving an AIDS-free generation and ending AIDS by 2030 may not be achievable [5,11,12].

Recognition of the impact of HIV-related discrimination in healthcare settings has brought to the forefront the urgent need to remodel education of the health workforce [13]. Theory suggests that specific training may result in better knowledge/skills of the health workforce when treating conditions of known high risk among the LGBT community and training may also reduce the stigma and discrimination of LGBT patients [14].

This systematic review assesses the effect of educational curricula and training for healthcare students and professionals on LGBT healthcare issues and offers a timely contribution to the debates about the role of professional educational interventions as the movement towards LGBT inclusion gains momentum globally.

## Methods

All primary research designed as trials (randomized, non-randomized controlled, pre–post) and qualitative studies in all languages were considered eligible for this review. There was no restriction based on year or country of publication.

Eligibility criteria were defined (see Table 1) using the PICOS approach which defines the population, intervention, comparator and outcomes relevant to the review [15].

**Table 1.** Eligibility criteria

Population	Medical doctors and dentists, nursing and midwifery professionals and pharmacists. Healthcare students studying for entry to one of the professions specified above
Interventions	All forms of training given to healthcare professionals on sexuality and LGBT specific health issues at undergraduate and postgraduate level
Comparator (if available)	Standard level training/No training on LGBT-specific issues
Outcome	Change in participants’ knowledge, attitude and or practice with regards to sexuality related issues and LGBT health

Following an initial review of keywords in relevant literature, the search terms, strategies and overall search process were defined. A detailed search strategy is in Appendix 1.

We searched the following databases; OVID Medline, PubMed, Web of Science, ScienceDirect, Education Resources Information Center (ERIC), TRIP, Google Scholar, Zetoc, Ebsco, CINAHL, PsycINFO. Cochrane library and University of York Centre for Reviews and Dissemination were also searched for ongoing systematic review protocols and published reviews on the subject of interest. Other sources of information used were University of Birmingham library, Ethos electronic thesis, ProQuest and grey literature online resource. The search was from the inception of the databases to 15 December 2015. Two researchers independently conducted the initial screening of titles and abstracts of articles identified through the search. The full-text articles were reviewed by three researchers for inclusion. The reference sections of included studies were screened to identify additional relevant studies.

A data extraction form was developed using the Cochrane consumers and communication review group’s data extraction template [16]. It was pilot tested prior to final use. The final version extracted information on the following: the author’s name, year and country of the study; the study design; type of population; characteristics of population; outcomes of interest; content of training; mode of delivery; time allotted for training; characteristics of the trainers; recommendations for future training. This process was carried out by two reviewers working independently.

A modified Downs and Black checklist [17] was used to assess the quality of non-randomized controlled studies and intervention studies without control. In grading the intervention studies without control, the following criteria were used: studies that scored ≥18 out of a maximum of 20 marks were graded as low risk, 15–17 moderate risk and <15 high risk. The Critical Appraisal Skills Programme checklist [18] was used for qualitative studies. Scores ≥8 out of a maximum of 10 were graded as low risk, 6–7 as moderate risk and <6 as high risk. Risk of bias assessment was undertaken by AS and checked by a second reviewer.

The review was registered on PROSPERO in March 2016 (CRD42016036430).

## Results

**Figure 1.**
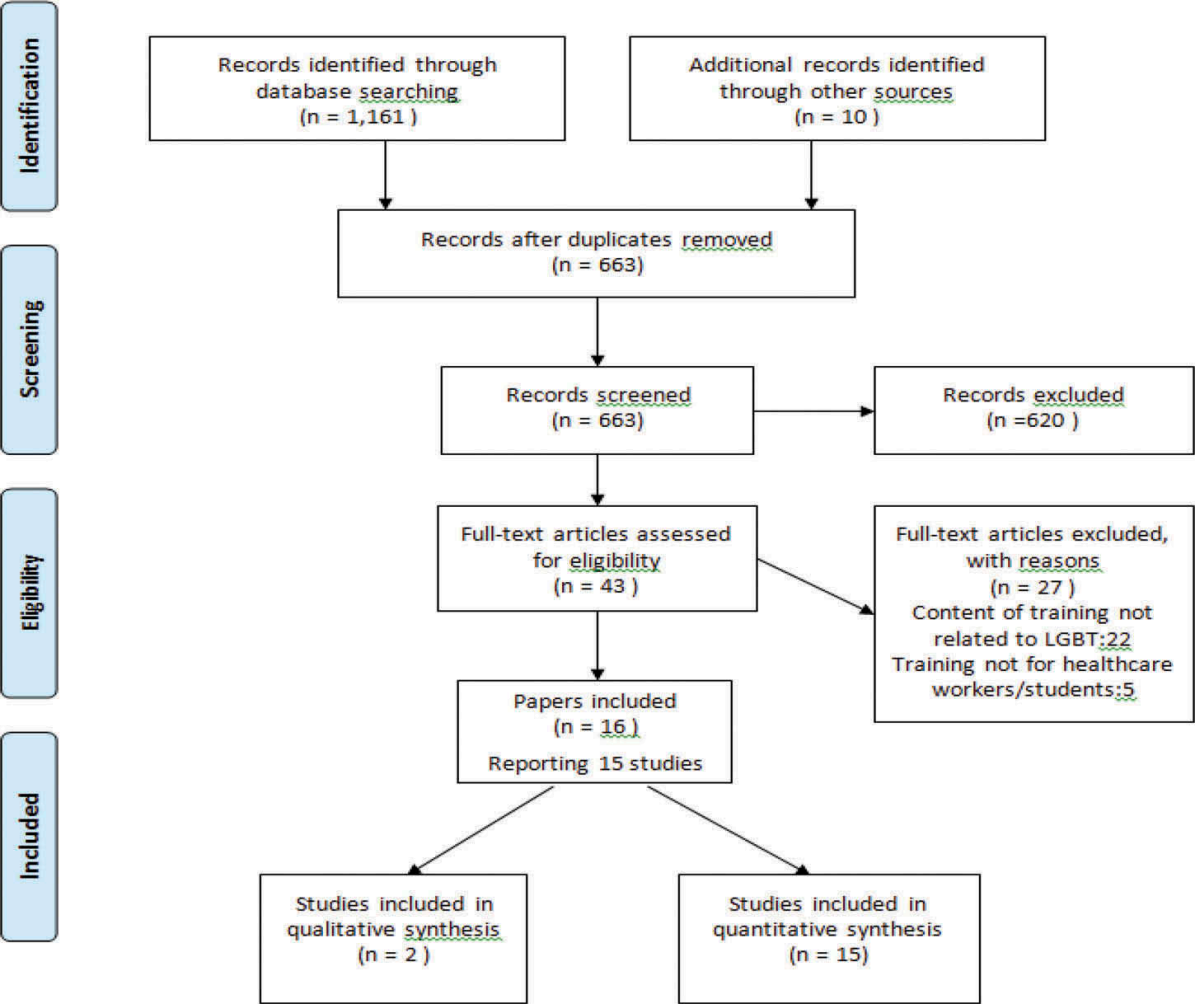
PRISMA diagram.

Searches identified 1171 studies. Removal of duplicates left 663 articles; 620 abstracts were excluded because they were not intervention studies. Of the remaining 43 abstracts, 27 were not eligible, 16 articles reporting 15 studies were eventually included. One study reported the quantitative and qualitative findings separately. The PRISMA flow diagram summarizes the included studies based on the eligibility criteria (Figure 1).

Ten studies had student populations: medical [19–25], nursing [26,27] and mixed population of students in nursing, pharmacy and the allied health professions [28]. Five studies presented data from healthcare service providers [29–33]: medical residents [29–31], practicing nurses [33] and one had a mixture of clinicians, nurses, counsellors and administrators [32]. Almost all studies took place in high-income western countries: 12 in the USA [19,20,22–24,26–31,33], 2 United Kingdom [21,25] and 1 in Kenya between 1977 and 2015. The Kenyan study used a mixed-method study design but published the quantitative and qualitative results separately [32,34]. Five articles were published from 1977 to 1989, while the remaining 11 were published after 2000. The sample size for the articles ranged from 13 [22] to 217 [25] subjects (see Table 2). Most of the articles in this review did not report the sex and age distribution of the participants.

### Study design

Three of the studies used a non-randomized pre/post design with concurrent comparators [19,22,28]. The remaining 12 studies had a pre/post intervention design without control; among this group, two used a mixed-method design [28,32]. Three of the quantitative studies had three months follow-up data [22,23,33]. One of the articles collected data using an online survey [26].

The qualitative element of the Kenyan study, published separately [34], used focus group discussion and framework approach while the second mixed-method study (USA) [28] used journal reflections and triangulated the findings with the quantitative results (Table 2).

### Risk of bias of included studies

Risk of bias is reported in Appendices 2–4.

The non-randomized controlled studies [19,21,22] were assessed to have high risk of bias due to confounding and moderate risk in selection of participants. In all cases, the control was students either in the same class or similar class who were not exposed to the sexuality-related courses offered as electives. The students were allowed to choose their electives based on preference. Two studies had low risk of bias due to missing data [19,21]. For measurement of outcomes, one study [22] did not have post intervention outcome data for the control group and was classified as having an unclear risk of bias, one study had a high risk [19] and the third had a low risk of bias [21].

For intervention studies without control, five were graded as low risk of bias [23,28–30,32], five as moderate risk [20,24,27,31,33] and two as high risk [25,26]. The articles did not provide enough information on the population included in the study, characteristics of subjects lost to follow-up and estimates of random variability for the main outcome. One of the articles reported on change in knowledge and attitude following the training but the data collection tool did not capture the same information pre and post, hence the result could not be compared [31]. The stand-alone qualitative study was classified as low risk [34], while the USA [28] qualitative study was classified as moderate risk.

### Training content

The content of the training can be grouped under the following five topics: key terms and terminology, stigma and discrimination, sexuality and sexual dysfunction, sexual history taking, LGBT-specific health and health disparities. The Kenyan study specifically addressed the health of MSM in sub-Saharan Africa [32,34]. All the training with the exception of two studies [20,29] involved multiple topics, the maximum reported in any study was four. Information was not available on the depth and extent to which the topics were discussed.

Eight of the articles reported teaching key terms and terminologies, mostly related to gay and lesbian terms [19–21,23–27]; these studies were mostly from interventions carried out over 10 years ago. Only four of the recent articles [23,26–28] talked about trans-related terminologies.

Stigma and discrimination related to expression of sexuality was discussed in eight studies [19,23,25–28,30,32]. The content ranged from stereotyping of sexual minority populations, cultural and religious bias in some communities to the emergence of new non-discriminatory policies based on the healthcare equality index in the USA [26].

In seven of the studies, the students were taught human sexuality [20–22,24,25,31,33]. This was often done in conjunction with disease states such as sexual dysfunction, sexual problems and STI/HIV. Sexual history taking was a key feature of training for medical residents and one of the commonly recurring topics in the reviewed studies; however, the effectiveness of this particular component of training was rarely assessed [19,21,22,28–31,33].

Healthcare issues specific to LGBT people were discussed under the following headings: HIV/AIDS and other STI [32,33], primary care issues [19,23,32], sexual dysfunction [25,31] and barriers to healthcare [30,31]. The study carried out in Kenya provided training on MSM-specific healthcare including mental health [32]. Transgender healthcare featured in three studies published in 2008 and 2015 [23,26,27]. LGBT health disparities featured in only two curricula [27,28].

### Trainers

In all but one study, the training was hosted and developed by universities and the facilitators/trainers were faculty in the institutions. The exception was the study carried out in Kenya where the training was carried out by an MSM counsellor, a community liaison officer, a social scientist, a senior research counsellor and two MSM who were members of a local non-governmental organization. In five studies, people from the LGBT community had been involved in the design or facilitated the training [19–21,23,27] (see Table 3).

**Table 2.** Summary of population characteristics and settings

Author (year)	Sample size	Type of student	Country	Study design	Follow-up
Bauman et al. (1985)	16	Medical student year 1	USA	Non-randomized control	Post-intervention
Carabez et al. (2015)		Nursing student	USA	Pre/post-intervention	Post-intervention
Carmichael et al. (1977)	104	Medical student year 2	USA	Pre/post-intervention	Post-intervention
Hawala-Druy et al. (2012)	106	Nursing, pharmacy, allied health students	USA	Pre/post-intervention Qualitative (journal reflections)	Post-intervention
Hawton et al. (1979)	42	Medical clinical student year 1	UK	Non-randomized control	Post-intervention
Johnson et al. (2015)	13	Medical student year 1	USA	Non-randomized control (post-intervention data not collected from the control group)	Post-intervention and three months
Kelly et al. (2008)	143	Medical student year 2	USA	Pre/post-intervention	Post-intervention
Loeb et al. (2010)	25	Medical residents	USA	Pre/post-intervention	Post-intervention
Mcgarry et al. (2002)	137	Medical residents	USA	Pre/post-intervention	Post-intervention
Rosen et al. (2006)	46	Medical residents	USA	Pre/post-intervention	Post-intervention
Strong et al. (2015)	88	Nursing student	USA	Pre/post-intervention	Post-intervention
Thomas et al. (1980)	145	Medical student year 2	USA	Pre/post-intervention	Post-intervention
Van der Elst et al. (2013)	74	Mixture healthcare workers	Kenya	Pre/post-intervention Qualitative (FGD)	Post-intervention and three months
Wylie et al. (2003)	217	Medical student year 4	UK	Pre/post-intervention	Post-intervention
Young et al. (1989)	200	Registered nurses	USA	Pre/post-intervention	Post-intervention and three months

UK: United Kingdom; USA: United States; FGD: focus group discussion.

**Table 3.** Summary table for training

Author, year and country	Topics	Hours allotted	Methods	Trainers
Bauman 1985 USA	Key terms and terminologies, stigma and discrimination, sexual history taking, LGBT health	11	Didactic lectures, small group discussions, social events, case review, role play	Faculty and LGBT people
Carabez 2014 USA	Key terms and terminologies, stigma and discrimination, sexual history taking	2	Didactic lectures, readings, instructions	Faculty
Carmichael 1977 USA	Sexuality and sexual dysfunction	10	Didactic lectures, social events, panel discussion, pre-reading	Faculty and LGBT people
Hawala-Druy 2012 USA	Stigma and discrimination, LGBT health	42	Didactic lectures, social events	Faculty
Hawton 1979 UK	Key terms and terminologies, sexuality and sexual dysfunction, sexual history taking	12	Didactic lectures, small group discussions, social events, role play	Faculty and LGBT people
Johnson 2015 USA	Sexuality and sexual dysfunction, sexual history taking, LGBT health	26	Didactic lectures, small group discussions, social events, shadowing, role play	Faculty
Kelly 2008 USA	Key terms and terminologies, stigma and discrimination, LGBT health	2	Small group discussions, patient panel	Faculty and LGBT people
Loeb 2010 USA	Sexual history taking	4	Case studies, role play	Faculty
McGarry 2002 USA	Key terms and terminologies, stigma and discrimination, sexual history taking, LGBT health	3	Didactic lectures, social events, case discussion, seminar	Faculty
Rosen 2006 USA	Sexuality and sexual dysfunction, sexual history taking	3	Didactic lectures, small group discussions, patient interview, panel discussion	Faculty
Strong 2015 USA	Key terms and terminologies, stigma and discrimination, LGBT health	1	Didactic lectures	Faculty and LGBT people
Thomas 1980 USA	Key terms and terminologies, sexuality and sexual dysfunction	34	Didactic lectures	Faculty
Van der Elst 2013 Kenya	Stigma and discrimination, LGBT health	16	Small group discussions, social events	Non-faculty and MSM
Wylie 2003 UK	Stigma and discrimination, sexuality and sexual dysfunction	24	Didactic lectures, small group discussions, seminar	Faculty
Young 1989 USA	Key terms and terminologies, sexuality and sexual dysfunction, sexual history taking	24	Didactic lectures, small group discussions, social events	Faculty

### Time allotted

A wide variation was reported in time allotted for the training ranging from 1 to 42 h. The median was 11 h. Six articles reported the use of 4 h or less [23,26,27,29–31] while in five studies more than 20 h was devoted to training [22,24,25,28,33]. It was impossible to make any useful deduction using time allotted for training and outcome of training in this review because the number and type of topics used in the training varied (see Table 3).

### Pedagogical method

Most curricula used multiple training methods. All but three [23,28,29] interventions delivered some content in the form of didactic lectures and two articles reported using only didactic lectures [24,27]. Other teaching and learning approaches reported by the articles were small group discussions followed by student presentations or summaries of group discussion [19,21–23,25,28,31–33]; social events (film and documentary screenings, educational games, multimedia presentations and social gathering) [19,20,22,28,30,32,33] and clinic based methods such as patient interviews, shadowing and case reviews [19,22,23,29–31]. Other methods less frequently used were role play [19,21,22,29], panel sessions [20,31], pre-reading of study materials [20] and seminars [25,30] (see Table 3).

### Quantitative outcome measures

Data available describe the direction of change in knowledge, attitude and practice of the subjects measured either directly or indirectly (see Table 4).

**Table 4.** Summary for outcomes

	Bauman	Carabez	Carmichael	Hawala-Druy	Hawton	Johnson	Kelley	Loeb	McGarry	Rosen	Strong	Thomas	Van der Elst	Wylie	Young
**Knowledge**															
Sexual health information					↑	↑								↑	
Sexual orientation		↑									↑				
Gender identity		↑									↑				
LGBT health							↑				↑		↑		
**Attitude**															
Accepting sexuality			↑		↑	↑						↑			
Masturbation			↑		↑							↑			
Homosexuality	↑		↑		↑		↑		→		↑	↑	↑		↑
Level of comfort and/cultural competence				↑			→		↑				↑		
**Practice**															
Sexual history documentation								↑							

↑: statistically significant improvement; →: no statistically significant change. Rosen did not capture the same data pre/post.

#### Knowledge

Seven studies measured change in participants’ knowledge regarding the following topics: sexuality and sexual dysfunction [21,22,25], LGBT health-related issues [22,27,32], key terms and terminologies [26]. Most of the studies did not teach the students about transgender health. All the studies reported a statistically significant improvement in knowledge immediately after the training and during the three months follow-up evaluation.

#### Attitude

Thirteen studies reported change in attitude focusing on accepting sexuality [20–22,24], masturbation [21,24,32], homosexuality [19–21,23,24,27,32,33] and level of comfort/cultural competence [28,30,32]. Development of a positive attitude towards homosexuality was the most measured component. Attitudes towards transgender people were not mentioned by the articles. The instruments for attitude varied, thereby making it impossible to determine which of the interventions was most effective regarding attitudinal change. However, all the articles documented a statistically significant increase in acceptance of LGBT people and sexuality-related issues except for one study which did not provide information [22].

In the non-randomized studies, pre-intervention attitudinal scores for the control group indicated negative attitude towards LGBT people.

#### Practice

One study assessed change in behaviour among medical residents in the USA. This was measured indirectly through the documentation and content of sexual history in patient charts. An overall improvement was reported, specifically with regards to current sexual activity, number of current sexual partners and gender of current sexual partners. However, documentation of gender of sexual partners over their lifetime, history of specific STIs and sexual behaviour were still judged to be inadequate post intervention [29]. None of the publications mentioned training participants to record the Sexual Orientation and Gender Identity of patients, in order to improve national electronic health records.

### Qualitative outcomes

The qualitative studies were based in the USA and Kenya.

Two main themes were identified from the qualitative studies: the process of changing values and attitudes to be more LGBT inclusive, and the constraints to the application of new values in practice.

#### Changing values and attitudes

Both qualitative publications note that, post intervention, participants talked about the changes that had taken place as a result of the information they received [28,34]. The US study gave examples of data where students initially held negative views (e.g. “My aunt left three daughters to live with another woman, please explain to me why? She loved me, I was so close to her but that is against my religious belief.”) but then modified their views and behaviours afterwards to be more inclusive, in this case re-contacting the estranged aunt. In the Kenyan study, participants were empowered to clarify their role and responsibilities as a professional, as being distinct from their role as an individual citizen, which was reflected in their attitude and practice in the workplace. As one of the participants from the study noted post-intervention:

As a clinician, my duty is to treat without imposing my values on the patient. That’s the positive thing I got from (the training program) and it’s what I’m doing now. [34]

#### Constraints to application of values in practice

Both studies (from the United States and Kenya) noted pre-existing cultural and religious prejudice against LGBT people or specifically MSM in African communities (“How can I accept them (LGBT)? I can still hear the drums from my church days” [28]. “MSM are unheard of in the place I come from” [34]); the experience of secondary stigma against the health facility from the community and against trained staff from professional colleagues (“You know MSM, as he had mentioned, are regarded as outcasts. Therefore, if you offer to treat them in your clinic, the community will perceive it as … the clinicians are also MSM” [34]); inadequate training of healthcare providers and lack of tools and guidelines to support staff (“Most of the medical personnel are not sensitized on issues to do with anal STIs and they are also not indicated in the STI charts” [34]). Finally, the Kenyan study noted that other pressures may limit the possibility to implement changes: “You may want to give the best, but the patients and the workload are too much” [34].

## Discussion

This review assessed all the studies published up to December 2015, which evaluated curricula and non-curricula-based training programmes for LGBT-related health, specifically for training healthcare students and postgraduate healthcare providers. A systematic review methodology was performed to enable a wide and thorough search of available studies and to extract and synthesize the study results in a robust way.

Some elements of measurement bias were observed in the studies. The measurement of outcomes did not cover all the topics that were taught in most of the studies and sexual history taking was rarely assessed. It is unclear how the authors made a decision on what was considered important enough to be measured. A huge gap, therefore, exists in determining the effectiveness of the interventions with regards to the various topics used for training.

Heterogeneity of topics in the training and the instruments used to measure outcomes precluded pooling of the results; hence, the efficacy of the training could not be ascertained in a meta-analysis. Our main finding is the lack of a unified conceptual model for training with regards to duration, the content, the time allotted and training methodology. The evidence is therefore inconclusive.

A reoccurring recommendation from the articles was the integration of sexual health and LGBT health into the main curriculum as compulsory block postings for medical, nursing and allied healthcare students at undergraduate and postgraduate level. This stems from the positive feedback received from attendees following training. This proposed integration would increase the number of teaching hours allotted, allow faculty to increase the number of topics and promote the use of a variety of teaching methodologies. All students would also be exposed, rather than the few who choose such courses as electives, thereby paving the way for a more rigorous curriculum evaluation.

From the review, topics on transgender health only featured in the more recent articles which is not surprising. LGBT health is a rapidly evolving field and a lot of providers are unfamiliar with the terminologies, protocols and recommendations for providing quality care. This could be one of the underlying factors preventing them from teaching their students. Two issues require addressing in order to identify the root causes of the deficiency in training: the availability of an integrated curriculum for teaching and the availability of competent faculty.

To impact the required knowledge, skills and attitudes needed to provide comprehensive LGBT healthcare, 16 topics have been recommended for medical colleges including chronic disease risk, unhealthy relationships, coming out, substance use, adolescent health, body image, transitioning and sex reassignment surgery [35,36]. However, the way these topics are used in curricula should be adapted to suit local context [37]. In our review, the maximum number of topics taught in any training was four which is inadequate to achieve competency. More broadly, a high proportion of medical schools in the USA have been reported to lack formal curricula for teaching sexual health related topics [38] while public health schools did not address comprehensive LGBT healthcare in their planned curriculum [39]. Although considerable improvement has occurred over the years with regards to the number of institutions and the content of sexual and LGBT health taught in medical schools [40], a disturbingly high proportion of medical students and practicing healthcare providers have received minimal or no training on LGBT health [41,42]. It is therefore not surprising that the authors of the studies included in this review consistently recommended that sexuality and LGBT healthcare courses should be mandatory to ensure that all healthcare students are exposed to the training.

In the last two decades, the time allotted for teaching LGBT-related topics has increased from a mean of 2 to 5 h in the USA [43,44]. The median recorded in this review was considerably higher and each of the interventions reported positive findings in relation to a short-term improvement in knowledge, attitude or practice.

Guidelines and training resources on LGBT healthcare [35,45–48] are available for healthcare providers to improve their knowledge and skills; however, they have not been rigorously evaluated. Although these resources may be used as part of a curriculum, they cannot be used as a complete substitution for a formally integrated competency-based training of the health workforce. Moreover, the reach of these resources is limited in low and middle-income countries. In some non-western countries, general attitudes are not inclusive or tolerant towards people who identify as LGBT [49]; this prejudice will influence the willingness to search for and use the resources to acquire knowledge and skills.

Five out of the 16 studies involved people from the LGBT community in the design or facilitation of training. It is likely that training is enhanced through the direct input of LGBT people who are likely to have a strong awareness of the barriers towards accessing healthcare within the LGBT community [36]. However, it may be a challenge to recruit LGBT people to participate in curriculum development and implementation in countries with LGBT criminalizing laws.

In countries with criminalizing laws, evidence of effective interventions to improve access to healthcare services for the LGBT population is scarce. In these countries, stand-alone clinics providing specialized services to the LGBT community exist, manned by specially trained competent service providers and maintained by developmental partners/donors. This strategy can only be a temporary solution. There is limited access because these clinics are unavailable in most cities and rural areas, they are expensive to run and therefore not sustainable without external funding. The staff and clients also run the risk of being targeted for violent acts by people who hold negative views of the LGBT population. They further exacerbate the social exclusion of sexual minorities by keeping them outside the formal health sector.

The only way to ensure equitable access to services for all is to train all healthcare providers to be culturally confident [23,26,50] and equip them with appropriate knowledge and skills [22,31]. The mainstream healthcare facilities and public health sector will then be able to provide competent and affirming care to clients and patients accessing services.

### Limitations of the existing literature

The majority of the studies were from the USA. The methodological quality of most of the study designs is weak and studies were at high risk of selection bias which may lead to more positive findings than in unselected populations. The long-term impact of curricula in terms of changes in attitude and actual translation of positive attitudes into clinical practice during patient contact is yet to be determined. Four of the studies were prior to 1990 and attitudes towards LGBT in westernized countries have changed since this time.

### Recommendations for further research

There is an urgent need for well-conducted studies evaluating LGBT health curricula, particularly in countries outside of the USA and where discrimination against LGBT people is high. There is a particular need for studies with a longer follow-up period to enable greater understanding of whether the short-term gains of LGBT health-related training that were evident from this review translate over the longer run. Future studies should use curricula that have been developed with input from national bodies and health professional training schools with input from LGBT community-based research institutes. Future research should evaluate practice, as well as knowledge and attitudes, and consider the importance of specific aspects of training including components relating to sexual health.

Finally, it was beyond the scope of this paper to address questions of educational theory – and the papers that met the inclusion criteria did not focus on this issue – but it could be extremely useful as this field develops, to use multidisciplinary approaches that explore and extend educational theory to complement evaluations of the effectiveness of educational interventions, so as to better understand why certain approaches work well for particular groups and particular contexts, and how these might change over time as societal attitudes change.

## Conclusion

This review found relatively few, generally low-quality studies where educational interventions were effective in improving knowledge, attitude and practice of healthcare students and professionals towards sexual health and LGBT health. However, it did identify potential components of effective educational interventions, which could be transferable to and adapted to different contexts. This is important given the urgent need to scale up access to good quality healthcare services to LGBT people globally and most especially in countries with laws that criminalize sexual minorities. However, the absence of good quality studies to inform decision makers on this crucial aspect of healthcare will delay this process and prolong the health disparities currently experienced by LGBT people.

## Appendix 1: Search terms

**Table:**

Homosexuality, Female/or Bisexuality/or Homosexuality/or Sexuality/or Minority Groups/or Homosexuality, Male/or Transgendered persons Sex Education/or Education, Medical/or Education/or Education, Medical, Undergraduate/or Education, Nursing/or Education, Dental/or Competency-Based Education/or Education, Medical, Continuing “Attitude of Health Personnel”/or Cultural Competency/or Clinical Competence Knowledge/or Health Knowledge, Attitudes, Practice Health Personnel/cl, ed. [Classification, Education]

## Appendix 2: Risk of bias for nonrandomized control studies

**Table:**

	Is the aim of the study clearly described?	Are the main outcomes to be measured clearly described in the introduction or methods section?	Bias due to confounding	Is confounding of the effect of intervention unlikely in this study?	Bias in selection of participants into the study	Did the authors describe how the population was selected?	Was selection into the study unrelated to intervention or outcome?	Are the characteristics of the population included in the study clearly described?	Did start of intervention and follow-up coincide for most subjects?	Bias in measurement of intervention	Is intervention well described?	Was information on intervention status recorded at the time of intervention?	Was information on intervention status unaffected by knowledge of outcome?	Bias due to missing data	Was intervention status complete for subjects in the intervention group	Were the characteristics of subjects lost to follow-up described?	Are outcome data complete?	Bias in measurement of outcomes	Was the outcome measure objective?	Were the statistical tests used to assess the main outcomes appropriate?	Are the findings presented clearly, objectively, and in sufficient detail to enable the reader to judge the results for himself/herself?	Did the investigators consider all possible logical interpretations of their results?
Bauman	Y	Y		N		Y	U	N	Y		Y	Y	Y		Y	U	Y		N	U	N	Y
Hawton	Y	Y		N		Y	U	Y	U		Y	Y	Y		Y	Y	N		N	Y	Y	Y
Johnson	Y	Y		N		Y	U	U	Y		Y	Y	Y		Y	Y	Y		N	U	U	U

## Appendix 3: Risk of bias for Pre/Post studies without control

**Table:**

	Is the aim of the study clearly described?	Are the main outcomes to be measured clearly described in the introduction or methods section?	Did the authors describe how the population was selected?	Were the subjects asked to participate in the study representative of the entire population from which they were recruited?	Are the characteristics of the population included in the study clearly described?	Are the interventions of interest clearly described?	Are the main findings of the study clearly described?	Does the study provide estimates of the random variability in the data for the main outcomes?	Have the characteristics of subjects lost to follow-up been described?	Have actual probability values been reported?	Were the statistical tests used to assess the main outcomes appropriate?	Are the findings presented clearly, objectively, and in sufficient detail to enable the reader to judge the results for himself/herself?	Did the investigators avoid introducing new results in the discussion?	Did the investigators consider all possible logical interpretations of their results?	Are the results adequately compared to previous studies in this area?	Are the conclusions clearly stated?	Are conclusions substantiated by the data that are presented in the results section?	Are generalizations confined to the population from which the sample was drawn?	Are the limitations of the study considered and were they taken into consideration when conclusions were drawn?	Are recommendations for future research made?
Cabarez	Y	Y	Y	Y	U	Y	Y	U	U	Y	Y	N	Y	N	Y	Y	Y	Y	N	Y
Carmichael	Y	Y	U	U	Y	Y	Y	Y	U	Y	Y	Y	Y	Y	N	Y	Y	Y	Y	Y
Hawala-Druy	Y	Y	Y	Y	Y	Y	Y	Y	U	Y	Y	Y	Y	Y	N	Y	Y	Y	Y	Y
Kelley	Y	Y	Y	Y	Y	Y	Y	Y	U	Y	Y	Y	Y	Y	N	Y	Y	Y	Y	Y
Loeb	Y	Y	Y	Y	Y	Y	Y	Y	U	Y	Y	Y	Y	Y	Y	Y	Y	Y	Y	Y
McGarry	Y	Y	Y	Y	Y	Y	Y	Y	Y	Y	Y	Y	Y	Y	Y	Y	Y	Y	Y	Y
Rosen	Y	Y	Y	Y	U	Y	Y	N	N	N	U	Y	Y	Y	N	Y	Y	Y	Y	Y
Strong	Y	Y	Y	N	Y	Y	Y	Y	N	Y	Y	Y	Y	Y	Y	Y	Y	Y	N	Y
Thomas	Y	Y	Y	Y	N	Y	Y	Y	N	Y	Y	Y	Y	N	Y	Y	Y	Y	N	Y
Van der Elst	Y	Y	Y	N	Y	Y	Y	Y	N	Y	Y	Y	Y	Y	Y	Y	Y	Y	Y	Y
Wylie	Y	Y	Y	Y	N	Y	Y	N	U	U	U	Y	Y	N	N	Y	Y	Y	N	N
Young	Y	Y	Y	Y	Y	Y	Y	N	N	Y	Y	Y	Y	Y	N	Y	Y	Y	Y	Y

## Appendix 4: Risk of bias for qualitative studies

**Table:**

	Does the study address a clearly focused research question	Was the choice of qualitative method appropriate	Was the research design appropriate to address the aim of the research	Was the recruitment strategy appropriate to the aims of the research	Was the data collected in a way that addressed the research issue	Has the relationship between researcher and participants been adequately considered	Have ethical issues been taken into consideration	Was the data analysis sufficiently rigorous	Is there a clear statement of findings	How valuable is the research
Hawala-Druy	Y	Y	Y	Y	Y	U	U	U	Y	U
Van der Elst	Y	Y	Y	Y	Y	U	Y	Y	Y	Y
